# Association between Sleep Onset Problem and Subjective Cognitive Complaints among Japanese Older Adults during the Coronavirus Disease 2019 Pandemic

**DOI:** 10.3390/ijerph20010156

**Published:** 2022-12-22

**Authors:** Yuriko Ikeda, Takayuki Tabira, Tadasu Ohshige, Tomomi Masumitsu, Hyuma Makizako, KU-OHL Project member

**Affiliations:** 1 Department of Occupational Therapy, School of Health Sciences, Faculty of Medicine, Kagoshima University, Kagoshima 890-8544, Japan; 2 Department of Physical Therapy, School of Health Sciences, Faculty of Medicine, Kagoshima University, Kagoshima 890-8544, Japan; 3 Department of Nursing, School of Health Sciences, Faculty of Medicine, Kagoshima University, Kagoshima 890-8544, Japan

**Keywords:** sleep quality, subjective cognitive complaints, older adults, community-dwelling, online survey

## Abstract

Older adults are more likely to have age-related sleep problems, which may result in the reduction of cognitive functions. This study was designed to examine the relationship between sleep onset problem and subjective cognitive complaints (SCC) among community-dwelling older adults during the coronavirus disease 2019 pandemic. In this study, 186 older adults aged 65 and above were enrolled and were instructed to respond to an online survey. This survey comprised questions regarding sleep quality (four items such as sleep duration, use of sleep medication), SCC (six domains), and sociodemographic information (eight items such as age, gender, stress condition). We classified the participants into two groups according to the presence or absence of sleep onset problem and examined the relationship between each SCC domain. The sleep onset problem (+) (n = 70) group had significantly higher frequency of scheduled memory decline, misplacement, disorientation in time, word recall decline, and forgetfulness. Furthermore, the sleep onset problem affected the participants’ scheduled memory after adjusted for potential covariates (OR, 2.28; 95%CI, 1.13–4.73; p = 0.02). Older adults with sleep onset problem may need to be evaluated for SCC and supported in term of both sleep status and SCC.

## 1. Introduction

The World Health Organization stated that coronavirus disease 2019 (COVID−19) can be characterized as a pandemic on 11 March 2020 [1]. In Japan, the state of emergency was first issued on 7 April 2020 because of the rapid increase in the number of infected individuals [2]. Individuals were required to restrict going out and practice social distancing to prevent the spread of infection. Additionally, it was clarified that many older adults living in the community refrain from going out and interacting with each other because of the fear of infection, and as a result, the frequency of daily activities, such as shopping and cooking, decreased [3]. 

Furthermore, it has been reported that older adults with subjective cognitive complaints (SCCs) or mild cognitive impairment (MCI), which are prodromal symptoms of dementia, experienced lifestyle changes that affect future cognitive decline because of COVID-19 [4]. Infection prevention measures have a great impact on the physical function, mental function, and lifestyle of older adults who are generally vulnerable, and particularly, these measures may have adverse effects on sleep [5,6,7]. It has been clarified that older adults have longer sleep latency, wake after sleep onset, experience early-morning awakening, have increased non-rapid eye movement sleep, and experience reduced rapid eye movement sleep [7,8]. Older adults are more likely to experience insomnia and sleep apnea because of stress and inactivity [9]. Short sleep times in healthy older adults may be associated with brain atrophy and cognitive decline [10], and age-related sleep problems are negatively associated with cognitive function and may increase the risk of developing dementia [11]. 

Several studies have examined the association between sleep and cognitive function. A systematic review of sleep and cognitive function in community-dwelling older adults suggested that the relationship between sleep and cognitive function was inconsistent; however, factors, such as depressive symptoms and sleep apnea, may have an effect [12]. Regarding SCC and sleep, poor sleep was related to forgetfulness [13], and there was also a clear relationship between items that appeal to SCC (i.e., memory, naming, and calculation) and the number of sleep problems [14]. Studies examining sleep quality and SCC categories reported a significant association between poor overall sleep quality and difficulty in subjective recalling location of objects, current memory loss, and decreased persistent attention; increased sleep latency was associated with difficulty in subjective recalling location of objects and increased frequency and severity of forgetfulness [15,16]. Furthermore, sleep onset problem is associated with decreased linguistic knowledge and long-term memory decline [17]. SCC are risk factors for MCI and dementia; moreover, these relationships may be stronger during the COVID−19 pandemics. Multifaceted and complex support, including sleep from SCC stages, is becoming more important. Understanding the status of sleep and cognitive function in community-dwelling older individuals under the COVID−19 pandemic and examining its relevance may help support these individuals after the pandemic. This study was designed to examine the association between sleep onset problem and SCCs among Japanese community-dwelling older adults.

## 2. Materials and Methods

### 2.1. Study Sample 

For this study, data were collected from an online survey panel by the Yahoo Japan Y cloud sourcing system. This survey was conducted as a preliminary survey of the Kagoshima University Online Health Laboratory (KU-OHL) [18] from 26 February 2021 to 27 February 2021. In total, 1602 Japanese adults aged 20 years and older responded to this online survey. Participants with stroke, Parkinson’s disease, dementia, or neurological disorders were excluded. Additionally, we excluded those who incorrectly answered questions that were identified as fraudulent responses (e.g., inconsistent answers or intentionally wrong answers) to ensure the reliability of the data. In this study, we included data on 200 older adults aged 65 years and above and analyzed 186 individuals excluding those who met the aforementioned exclusion criteria. The content of the questionnaire survey was developed by 18 members of the KU-OHL, which comprised medical doctors, nurses, physical therapists, and occupational therapists. 

### 2.2. Ethical Approval

This study was conducted according to the Declaration of Helsinki, and the study protocol was approved by the Ethics Committee of the Faculty of Medicine, Kagoshima University (200240). Informed consent was obtained from all participants in this study.

### 2.3. Measurements

#### 2.3.1. Sleep Quality

Four simple questions were used to obtain information about sleep. To assess sleep duration, the participants were asked “How long do you usually sleep?” and answered their sleep duration in 10-min intervals. To assess sleep quality, the participants were asked two questions—“Can you have a refreshing awakening in the morning?” and “Do you have sleep onset problem?”—and answered these questions on a four-point Likert scale (i.e., “always,” “sometimes,” “not much,” and “not at all”). To evaluate whether the participants took medications for sleeping, they were asked “Do you take medicine to sleep?” and answered the question using a four-point Likert scale (“not at all,” “less than once a week,” “1–2 times a week,” and “3 times or more a week”). Participants who answered “always” or “sometimes” for the question “Do you have sleep onset problem?” were classified into the sleep onset problem (+) group, whereas those who answered “not much” or “not at all” were classified into the sleep onset problem (−) group.

#### 2.3.2. SCCs 

SCCs were investigated using six items: scheduled memory decline (Do you ever forget your appointment?), misplacement (Do you have difficulty remembering where you leave objects like a wallet or a key?), disorientation in place (Do you have difficulty recognizing familiar palaces (e.g., road or supermarket)?), disorientation in time (Do you have difficulty recognizing what month and day it is today?), word recall decline (Do you have difficulty recalling what you want to say?), and forgetfulness (Do you consider yourself as being forgetful?). The participants answered each question on a three-point Likert scale (“always,” “sometimes,” and “not at all”) [19,20].

#### 2.3.3. Sociodemographic Variables

The participants were instructed to provide their age, gender, education level, living situation, stress condition, physical activity, alcohol consumption, job status, and history of depression. The stress condition was confirmed by answering on a four-point Likert scale (“a lot,” “sometimes,” “not so much,” and “not at all”) to the question “Did you feel stressed in the past year?”. Physical activity was measured using the International Physical Activity Questionnaire (IPAQ) [21], participants were divided into three levels: low, moderate, and high. Alcohol consumption was based on the amount equivalent to about 20 g of pure alcohol per day (sake; 180 mL) recommended by the Ministry of Health, Labor and Welfare [22]. There were nine options for job status. In this study, the participants were divided into two categories: employed (e.g., office worker, self-employed, part-time job) and unemployed. Depression was confirmed based on past and current medical history. Depression was excluded from the analysis because only three had a history of depression.

### 2.4. Statistical Analysis

The characteristics, sleep quality, and SCCs of the participants were compared between the sleep onset problem (+) and sleep onset problem (−) groups. Student’s *t*-test and Pearson’s chi-square test were used for continuous variables (e.g., age and sleep duration) and categorical variables (e.g., a refreshing awakening in the morning and scheduled memory decline), respectively. A multiple logistic analysis was performed to examine the association between sleep onset problem and SCC. The presence of sleep onset problem was set as the dependent variable, and five items for SCCs that were significantly different between the two groups were set as the independent variables. Two models were developed: models with and without adjustment for covariates (i.e., age, gender, education, living situation, stress condition, IPAQ class and alcohol consumption, job status). In addition, we performed subgroup analyses by gender, as we found differences in the proportion of male and female with and without sleep onset problem. Our findings confirmed that all variance inflation factors were less than 10 and no multicollinearity existed. All statistics analyses were performed using R version 4.1.1 (R core team) [23] with the significance level set at 5%.

## 3. Results

Of the 186 participants, 70 (37.5%) were in sleep onset problem (+) group and 116 (62.3%) were in sleep onset problem (−) group. The sociodemographic characteristics of the participants with and without sleep onset problem are presented in Table 1. The sleep onset problem (+) group was significantly older (*p* = 0.003, d = 0.46) than sleep onset problem (−) group, but the actual age difference was only 1.9 years. Regarding the proportion of gender in each group, both groups had a higher number of male participants, but the sleep onset problem (+) group had a higher proportion of female than the sleep onset problem (−) group (*p* = 0.001, φ = 0.26). In addition, the group with sleep onset problem had more stress than the group without (*p* = 0.004, V = 0.27). See Supplementary Table S1 for results of subgroup analyses of sociodemographic characteristics in male and female.

Table 2 shows the results of the comparison of sleep quality and SCCs between the two groups. Regarding sleep quality, the sleep onset problem (+) group experienced significantly fewer refreshing awakenings in the morning (*p* < 0.001, V = 0.50) and took medication for sleeping more often (*p* < 0.001, V = 0.35) than the sleep onset problem (−) group. Regarding SCCs, the frequencies of scheduled memory decline (*p* < 0.001, V= 0.33), misplacement (*p* = 0.03, V= 0.19), disorientation in time (*p* = 0.001, V = 0.28), word recall decline (*p* = 0.018, V= 0.21), and forgetfulness (*p* < 0.001, V = 0.30) were higher in the sleep onset problem (+) group than in the sleep onset problem (−) group. Supplementary Table S2 showed the results of subgroup analyses of sleep quality and SCCs in male and female. The male group with sleep onset problem had significantly higher frequency of scheduled memory decline, disorientation in time, word recall decline, and forgetfulness than the male group without sleep onset problem. On the other hand, the female group with sleep onset problem had significantly higher frequency of scheduled memory decline and misplacement than the female group without sleep onset problem.

Table 3 shows the results of the multiple logistic analysis for sleep onset problem and SCCs. The crude model (the model without adjustment for covariates) demonstrated that sleep onset problem was associated with scheduled memory decline (odds ratio (OR) = 2.06; 95% confidence interval (CI) = 1.08–4.05; *p* = 0.03) and forgetfulness (OR = 2.17; 95% CI = 1.03–4.77; *p* = 0.04). The model with adjustment for covariates demonstrated that sleep onset problem was associated with scheduled memory decline (OR = 2.28; 95% CI = 1.13–4.73; *p* = 0.02).

Akaike information criterion (AIC): crude model: 232.3, adjusted model: 220.9

Table 4 shows the results of the multiple logistic analysis for sleep onset problem and SCCs in the male and female groups, respectively. There was no association between sleep onset problem and SCCs for each group.

## 4. Discussion

This study examined the association between sleep onset problem and SCCs in Japanese community-dwelling older adults during the COVID-19 pandemic using an online survey. As a result, it was clarified that sleep onset problem related to the decline in scheduled memory.

The psychological stress such as the pandemic causes insomnia, and restrictions on going out and lifestyle changes to prevent the spread of infection may lead to problems with the rhythm of daily life. Before the COVID-19 pandemic, a large-scale objective sleep data survey involving Japanese individuals showed that the average sleep time for older adults in their 60s and 70s was approximately 7 h, and sleep latency was 14–16 min; moreover, these individuals took longer to fall asleep than younger individuals [24]. The problems of sleep disorders and hypnotic use in Japanese adults (7.5–8.7% of older adults in their 60s and 70s) have been highlighted in the past [25]. Approximately 20% of older adults aged more than 60 years experience sleep onset problem at least once a week and have been reported to be associated with mental health decline [26]. In this study, the average sleep time of the subjects was approximately 7 h; among the study participants, 37.5% complained of sleep onset problem, and approximately 9.7% used sleep medications. No difference in sleep time was observed between this study and previous studies; however, the results of this study showed that more older adults had sleep onset problem and used sleep medications. In addition, males with sleep onset problem were more likely to use sleep medication more frequently. We speculate that this result is influenced by the pandemic; however, their direct causal relationship is unknown. There is no denying that the COVID-19 pandemic has increased sleep onset problem in older adults. 

In a long-term follow-up survey, it has been shown that sleep quality problems in middle-aged individuals contribute to cognitive decline in later years [27], and sleep quality was a risk factor for future MCI (OR = 2.67) and Alzheimer’s disease (OR = 2.81) [28]. Another self-reported study reported that sleep onset problems increased the risk of dementia (OR = 1.49) [29]. In contrast, SCCs are risk factors for MCI and dementia, and carefully observing the complaints of older adults is necessary. The prevalence of SCCs among community-dwelling older adults varies. Studies presenting screening results for SCCs reported that 64% of the participants had forgetfulness, 68% had difficulty remembering places, and 52% had scheduled memory problems [30]. In this study, community-dwelling older adults frequently felt forgetfulness (78.4%), experienced word recall decline (61.2%), had misplacements (51.6%), and had scheduled memory decline (41.9%). Although comparing this study with previous studies is difficult because of potential factors, such as the evaluation method or differences in environmental factors, it was shown that many older adults had forgetfulness, in the COVID-19 pandemic. Furthermore, sleep onset problem was shown to relate to scheduled memory decline. Scheduled memory is an advanced cognitive function and is one of the functions that tend to encounter problems from an early stage [31,32]. In previous studies, sleep onset problem has been shown to be associated with decreased linguistic knowledge and long-term memory decline [17]; however, we believe that we should consider the effects of sleep onset problem on scheduled memory. On the other hand, subgroup analyses revealed that males with sleep onset problem, in particular, reported SCCs more frequently, but no association was found between sleep onset problem and SCCs. Sleep quality and SCC may need to be carefully examined in males.

This study has several limitations. First, this study adopted a cross-sectional study design and could not investigate changes in sleep status and cognitive function before and after the COVID-19 pandemic. The effects of physical and psychological stress caused by the pandemic on lifestyle could not be considered. Second, because an online survey was used in this study, participants were likely to be older adults with good internet access, have a high level of education and more males than in surveys of ordinary older adults. In particular, education levels among the participants in this study (64.5% had an education level of 12 years or more) were clearly higher than among older adults in general (over 12 years: 38.2% in their 60s and 21.5% in their 70s) [33]. These selection biases may have affected the results. In addition, online surveys make it difficult to generalize results. Finally, we have not investigated the sleep characteristics of the older adults in this study, such as wake after sleep onset, early-morning awakening, and daytime sleepiness, and we could not take a more detailed consideration. Further detailed investigation is required.

## 5. Conclusions

In this study, we conducted an online survey among community-dwelling older adults during the COVID-19 pandemic to examine the relationship between sleep quality and SCC. The results showed that among older adults with good Internet access and higher education, those with sleep disorders had significantly more SCC than those without. This trend was particularly pronounced among males. Furthermore, it was shown that sleep onset problem is associated with scheduled memory. On the other hand, it is a concern that there is no association between sleep onset problem and SCCs in each of the male and female groups. Nevertheless, we propose that we should also evaluate SCC (especially scheduled memory) for older adults who have sleep onset problem, and approach from both sleep problem and cognitive function.

## Figures and Tables

**Table 1:** Characteristics of participants.

Parameter	Total *n* = 186	Sleep Onset Problem		*p*-Value	Effect Size
		*n* = 70	(−) *n* = 116		
Age(years), mean (SD)	69.7 (4.2)	70.9 (4.6)	69.0 (3.8)	0.003 ^a^	d = 0.46
Gender, *n* (%)					
Male	130 (70.3)	38 (54.3)	92 (79.3)	0.001 ^b^	φ = 0.26
Female	55 (29.7)	31 (44.3)	24 (20.7)		
Education, *n* (%)					
<12years	5.0 (2.6)	3 (4.3)	2 (1.7)	0.21 ^b^	V = 0.12
12years	61 (32.8)	27 (38.6)	34 (29.3)		
>12years	120 (64.5)	40 (57.1)	80 (69.0)		
Living situation, *n* (%)					
Alone	21 (11.3)	7 (10.0)	14 (12.1)	0.85 ^b^	V = 0.03
With others	165 (88.7)	63 (90.0)	102 (87.9)		
Stress condition, *n* (%)					
A lot	30 (16.1)	15 (21.4)	15 (12.9)	0.004 ^b^	V = 0.27
Sometimes	83 (44.6)	39 (55.7)	44 (37.9)		
Not so much	59 (31.7)	14 (20.0)	45 (38.8)		
Not at all	14 (7.5)	2 (2.9)	12 (10.3)		
IPAQ level, *n* (%)					
law	81 (43.6)	33 (47.1)	48 (41.4)	0.21 ^b^	V = 0.13
Moderate	57 (30.6)	24 (34.3)	33 (28.4)		
high	48 (25.8)	13 (18.6)	35 (30.2)		
Alcohol amount, *n* (%)					
≦180 mL/day	125 (67.2)	50 (71.4)	75 (64.7)	0.43 ^b^	φ = 0.07
>180 mL/day	61 (32.8)	20 (28.6)	41 (35.3)		
Job status, *n* (%)					
Employed	72 (38.7)	26 (37.1)	46 (36.9)	1.00 ^b^	φ = 0.09
Unemployed	114 (61.3)	44 (62.9)	70 (63.1)		

IPAQ; International Physical Activity Questionnaire, ^a^ Student’s *t*-test. ^b^ Pearson’s chi-square. d; Cohen’s d, φ; phi coefficient, V; Cramer’s V.

**Table 2:** Comparison of sleep duration and quality, and SCC between the participant with and without sleep onset problem.

Parameter	Total *n* = 186	Sleep Onset Problem		*p*-Value	Effect Size
		(+) *n* = 70	(−) *n* = 116		
Sleep duration, min/day, mean (SD)	429.3 (63.2)	426.1 (69.3)	431.2 (59.3)	0.59 ^a^	d = 0.08
A refreshing awakening in the morning, *n* (%)					
Always	90 (48.4)	13 (18.6)	77 (66.4)	0.001 ^b^	V = 0.50
Sometimes	65 (34.9)	35 (50.0)	30 (25.9)		
Not much	26 (13.9)	20 (28.6)	6 (5.2)		
Not at all	5.0 (2.7)	2 (2.9)	3 (2.6)		
Use of sleep medications, *n* (%)					
Not at all	168 (90.3)	54 (77.1)	114 (98.3)	*p* < 0.001 ^b^	V = 0.35
Less than once a week	4.0 (2.2)	3 (4.3)	1 (0.9)		
1–2 times a week	4.0 (2.2)	4 (5.7)	0 (0.0)		
3times or more a week	10 (5.3)	9 (12.9)	1 (0.9)		
Scheduled memory decline, *n* (%)					
Always	9.0 (4.8)	5 (7.1)	4 (3.4)	*p* < 0.001 ^b^	V = 0.33
Sometimes	69 (37.1)	39 (55.7)	30 (25.9)		
Not at all	108 (58.1)	26 (37.1)	82 (70.7)		
Misplacement, *n* (%)					
Always	11 (5.9)	5 (7.1)	6 (5.2)	0.03 ^b^	V = 0.19
Sometimes	85 (45.7)	40 (57.1)	45 (38.8)		
Not at all	90 (48.4)	25 (35.7)	65 (56.0)		
Disorientation in place, *n* (%)					
Always	1.0 (0.5)	0 (0.0)	1 (0.9)	0.65 ^b^	V = 0.07
Sometimes	7.0 (3.8)	2 (2.9)	5 (4.3)		
Not at all	178 (95.7)	68 (97.1)	110 (94.8)		
Disorientation in time, *n* (%)					
Always	3.0 (1.6)	1 (1.4)	2 (1.7)	0.001 ^b^	V = 0.28
Sometimes	37 (19.9)	24 (34.3)	13 (11.2)		
Not at all	146 (78.5)	45 (64.3)	101 (87.1)		
Word recall decline, *n* (%)					
Always	18 (9.7)	8 (11.4)	10 (8.6)	0.018 ^b^	V = 0.21
Sometimes	96 (51.6)	44 (62.9)	52 (44.8)		
Not at all	72 (38.7)	18 (25.7)	54 (56.6)		
Forgetfulness, *n* (%)					
Always	21 (11.3)	11 (15.7)	10 (8.6)	*p* < 0.001 ^b^	V = 0.30
Sometimes	125 (67.2)	55 (78.6)	70 (60.3)		
Not at all	40 (21.5)	4 (5.7)	36 (31.0)		

^a^ Student’s *t*-test. ^b^ Pearson’s chi-square. d; Cohen’s d, V; Cramer’s V.

**Table 3:** Association between the sleep onset problem and subjective cognitive complaints.

	Crude Model			Adjusted Model		
	OR	95%CI	*p*-Value	OR	95%CI	*p*-Value
Scheduled memory decline	2.06	1.08–4.05	0.03	2.28	1.13–4.73	0.02
Misplacement	0.84	0.43–1.62	0.60	0.98	0.47–2.01	0.95
Disorientation in time	1.89	0.89–4.09	0.09	1.64	0.70–3.93	0.25
Word recall decline	1.04	0.58–1.85	0.89	1.02	0.54–1.91	0.94
Forgetfulness	2.17	1.03–4.77	0.04	1.91	0.85–4.51	0.12

OR: odds ratio. CI: confidence interval. Adjusted model; age, gender, education, living situation, stress condition, International Physical Activity Questionnaire class, alcohol amount, job status.

**Table 4:** Association between the sleep onset problem and subjective cognitive complaints in male group and female group.

	Male						Female					
	Crude Model			Adjusted Model			Crude Model			Adjusted Model		
	OR	95%CI	*p*-Value	OR	95%CI	*p*-Value	OR	95%CI	*p*-Value	OR	95%CI	*p*-Value
Scheduled memory decline	1.70	0.73–3.98	0.21	2.12	0.79–5.78	0.13	2.00	0.69–6.36	0.21	2.03	0.67–6.67	0.21
Misplacement							1.63	0.52–5.46	0.41	1.67	0.49–6.01	0.42
Disorientation in place												
Disorientation in time	1.61	0.57–4.73	0.37	2.31	0.63–9.36	0.21						
Word recall decline	1.74	0.84–3.67	0.14	1.77	0.73–4.46	0.21						
Forgetfulness	178	0.70–4.60	0.22	2.00	0.66–6.41	0.22						
Akaike information criterion (AIC)	149.3			133.8			75.7			85.4		

OR: odds ratio. CI: confidence interval. Adjusted model; age, gender, education, living situation, stress condition, International Physical Activity Questionnaire class, alcohol amount, job status.

## Data Availability

There are no linked research datasets for this study. The authors do not have permission to share the data.

## Supplementary Materials

The following supporting information can be downloaded at: https://www.mdpi.com/article/10.3390/ijerph20010156/s1, Table S1: Characteristics of participants in male group and female group; Table S2: Comparison of sleep duration and quality, and SCC between the participant with and without sleep onset problem in male group and female group.
