# Supplementary material for: Association between Sleep Onset Problem and Subjective Cognitive Complaints among Japanese Older Adults during the Coronavirus Disease 2019 Pandemic
Source: Int J Environ Res Public Health. 2022 Dec 22;20(1):156. doi: 10.3390/ijerph20010156 (PMC9819132; doi:10.3390/ijerph20010156)
Supplement: Supplementary file 1 [file ijerph-20-00156-s001.zip › ijerph-2078804-supplementary.pdf]

Table S1 Characteristics of participants in male group and female group

parameter	male			female		
	sleep onset problem		p-value (Effect size)	sleep onset problem		p-value (Effect size)
	(+) *n*=38	(-) *n*=92		(+) *n*=31	(-) *n*=24	
Age(years), mean (SD)	70.3(4.4)	68.7(3.5)	0.03[a] (d=0.42)	71.6(4.8)	70.2(4.6)	0.52[a] (d=0.30)
Education, *n* (%)						
>12 years	3(7.9)	1(1.6)	0.08[b] (V=0.19)	0(0.0)	1(4.1)	0.52[b] (V=0.16)
12years	12(31.6)	23(25.3)		15(48.4)	11(45.8)	
<12years	23(60.5)	67(73.6)		16(51.6)	12(50.0)	
Living situation, *n* (%)						
Alone	4(10.5)	9(9.8)	1.0[b]	3(9.7)	5(20.8)	0.44[b]
With others	34(89.5)	83(90.2)	(φ=0.01)	28(90.3)	19(79.2)	(φ=0.16)
Stress condition, *n* (%)						
A lot	6(15.8)	10(10.9)		9(29.0)	5(20.8)	
Sometimes	20(52.6)	21(33.1)	0.07[b]	19(61.3)	13(54.2)	0.44[b]
Not so much	11(28.9)	40(43.5)	(V=0.23)	2(6.5)	5(20.8)	(V=0.22)
Not at all	1(2.6)	11(12.0)		1(3.2)	1(4.2)	
IPAQ level, *n* (%)						
law	14(36.8)	35(38.0)	0.11[b] (V=0.18)	18(58.1)	13(54.2)	0.85[b] (V=0.08)
Moderate	17(44.7)	26(28.3)		7(22.6)	7(29.2)	
high	7(18.4)	31(33.7)		6(19.4)	4(16.7)	
Alcohol amount, *n* (%)						
≦180ml/day	20(52.6)	53(57.6)	0.75[b]	29(93.5)	22(91.7)	1.0[b]
＞180ml/day	18(47.4)	39(42.4)	(φ=0.05)	2(6.5)	2(8.3)	(φ=0.04)
Job status, *n* (%)						
employed	20(52.6)	42(42.5)	0.39[b]	6(19.4)	4(16.7)	1.0[b]
unemployed	18(47.4)	50(57.5)	(φ=0.09)	25(80.6)	20(83.3)	(φ=0.04)

IPAQ; International Physical Activity Questionnaire, [a] Student's t-test. [b] Pearson's chi-square, d; Cohen's d, φ; phi coefficient, V; Cramer's V

Table S2 Comparison of Sleep duration and quality, and SCC between the participant with and without sleep onset problem in male group and female group

**parameter**	**male**			**female**		
	**sleep onset problem**		**p-value (Effect size)**	**sleep onset problem**		**p-value (Effect size)**
	**(+) *n*=38**	**(-) *n*=92**		**(+) *n*=31**	**(-) *n*=24**	
Sleep duration, min/day, mean (SD)	431.8(78.9)	434.3(58.4)	0.84[a] (d=0.04)	419.7(58.7)	419.6(63.1)	0.99[a] (d=0.03))
A refreshing awakening in the morning, *n* (%)						
Always	8(21.1)	61(66.3)		5(16.1)	16(66.7)	
Sometimes	18(47.4)	24(26.1)	<0.001[b]	17(54.8)	6(25.0)	0.002[b]
Not much	11(28.9)	4(4.3)	(V=0.46)	8(25.8)	2(8.3)	(V=0.52)
Not at all	1(2.6)	3(3.3)		1(3.2)	0(0.0)	
Use of sleep medications, *n* (%)						
Not at all	31(81.6)	91(98.9)		23(74.2)	23(95.8)	
Less than once a week	0(0.0)	0(0.0)	0.001[b]	3(9.7)	1(4.2)	0.16[b]
1~2times a week	1(2.0)	0(0.0)	(V=0.39)	2(6.5)	0(0.0)	(V=0.34)
3times or more a week	6(15.8)	1(1.1)		3(9.7)	0(0.0)	
Scheduled memory decline, *n* (%)						
Always	1(2.6)	2(2.2)	0.004[b]	4(12.9)	2(8.3)	0.03[b]
Sometimes	22(57.9)	25(27.2)	(V=0.29)	16(51.6)	5(20.8)	(V=0.36)
Not at all	15(39.5)	65(70.7)		11(35.5)	17(70.8)	
Misplacement, *n* (%)						
Always	2(5.3)	4(4.3)	0.28[b]	2(6.5)	2(8.3)	0.02[b]
Sometimes	22(57.9)	40(43.5)	(V=0.14)	18(58.1)	5(20.8)	(V=0.38)
Not at all	14(36.8)	48(52.2)		11(35.5)	17(70.8)	
Disorientation in place, *n* (%)						
Always	0(0.0)	1(1.1)	0.43[b]	0(0.0)	0(0.0)	1.00[b]
Sometimes	0(0.0)	3(3.3)	(V=0.12)	2(6.5)	2(8.3)	(V=0.04)
Not at all	38(100)	89(95.7)		29(93.5)	22(91.7)	
Disorientation in time, *n* (%)						

Always	0(0.0)	2(2.2)	<0.001[b] (V=0.36)	1(3.2)	0(0.0)	0.59[b] (V=0.14)
Sometimes	12(31.6)	5(5.4)		12(38.7)	8(33.3)	
Not at all	26(68.4)	85(92.4)		18(58.1)	16(66.7)	
Word recall decline, *n* (%)						
Always	7(18.4)	6(6.5)	0.004[b] (V=0.29)	1(3.2)	4(16.7)	0.18[b] (V=0.25)
Sometimes	23(60.5)	40(43.5)		21(67.7)	12(50.0)	
Not at all	8(21.1)	46(50.0)		9(29.0)	8(33.3)	
Forgetfulness, *n* (%)						
Always	6(15.8)	7(7.6)	0.003[b] (V=0.29)	4(12.9)	3(12.5)	0.15[b] (V=0.26)
Sometimes	30(78.9)	55(59.8)		25(80.9)	15(62.5)	
Not at all	2(5.3)	30(32.6)		2(6.5)	6(25.0)	

[a] Student's t-test, [b] Pearson's chi-square, d; Cohen's d, φ; phi coefficient, V; Cramer's V
